# Zika Virus Infection in Tourists Travelling to Thailand: Case Series Report

**DOI:** 10.3390/tropicalmed6010003

**Published:** 2020-12-28

**Authors:** Natàlia Romaní, Marie Antoinette Frick, Elena Sulleiro, Carlota Rodó, María Espiau, Diana Pou, Aroa Silgado, Anna Suy, Tomás Pumarola, Pere Soler-Palacín, Antoni Soriano-Arandes

**Affiliations:** 1 Pediatric Infectious Diseases and Immunodeficiencies Unit, Hospital Universitari Vall d’Hebron, Vall d’Hebron Research Institute, Universitat Autònoma de Barcelona, 08035 Barcelona, Spain; nromani@vhebron.net (N.R.); mafrick@vhebron.net (M.A.F.); mespiau@vhebron.net (M.E.); psoler@vhebron.net (P.S.-P.); 2 Department of Microbiology, Hospital Universitari Vall d’Hebron, 08035 Barcelona, Spain; esulleir@vhebron.net (E.S.); aroasilgado@gmail.com (A.S.); tpumarola@vhebron.net (T.P.); 3 Maternal Fetal Medicine Unit, Department of Obstetrics, Hospital Universitari Vall d’Hebron, Vall d’Hebron Barcelona Hospital Campus, Passeig Vall d’Hebron 119-129, 08035 Barcelona, Spain; crodo@vhebron.net (C.R.); asuy@vhebron.net (A.S.); 4 Center of International Health and Transmissible Diseases Drassanes-Vall d’Hebron, Hospital Universitari Vall d’Hebron, 08035 Barcelona, Spain; d.pou@vhebron.net

**Keywords:** zika virus, zika virus infection, mosquito-borne disease, travel, travel-associated, children, neonate, mother-to-child transmission, Thailand

## Abstract

Thailand is a popular tourist destination where Zika virus (ZIKV) transmission is currently active. To our knowledge, there are no reports of ZIKV infection imported from Thailand and affecting children. Here, we describe the clinical and microbiological findings in three cases of vector-borne ZIKV infection: An 11-year-old boy, a 2-year-old girl, and her pregnant mother, this last case leading to the prenatal exposure of her second baby to ZIKV in the second trimester of pregnancy. All patients were diagnosed after traveling to Thailand between September 2019 and January 2020. No complications were detected in any patient at follow-up, and the prenatally exposed fetus showed no abnormalities during intensive antenatal health care monitoring. On postnatal study, there were no clinical signs or microbiological findings of mother-to-child ZIKV transmission. ZIKV IgG was initially positive, but seroreversion occurred at 4 months of life. This report describes the clinical and serological evolution of vector-borne ZIKV infection occurring in dengue-naïve tourists returning from Thailand. The World Health Organization currently recommends that pre-travel advice to prevent arbovirus infection should be maintained in travelers to Southeast Asia.

## 1. Background

Zika virus (ZIKV) is a mosquito-borne flavivirus related to the dengue, yellow fever, and West Nile viruses [1,2,3]. ZIKV infection during pregnancy has been associated with adverse fetal outcomes, such as congenital microcephaly and other neurodevelopmental abnormalities [4]. Outside the prenatal period, ZIKV infection in humans is mainly asymptomatic. Mild, self-limiting symptoms such as pruritic maculopapular rash, fever, myalgia, arthralgia, and headache occur in approximately 20% of infected individuals [3,5,6]. Complications such as Guillain-Barré syndrome and meningitis can occur, but are rare in the pediatric age. Antibody cross-reactivity with other mosquito-borne flaviviruses during testing and overlapping of the clinical features can lead to a misdiagnosis of ZIKV [3,7].

Although cases of ZIKV infection have been reported in travelers to Thailand (13 cases in 2013–2019) [8,9,10,11], we encountered no descriptions of this infection in the pediatric population or in prenatally exposed newborns. In this article, we describe two pediatric cases of ZIKV infection and a case of prenatal exposure in tourists travelling to Thailand between September 2019 and January 2020.

## 2. Case Descriptions

### 2.1. Rash in a Child on a Flight Returning from Thailand

**Figure 1:**
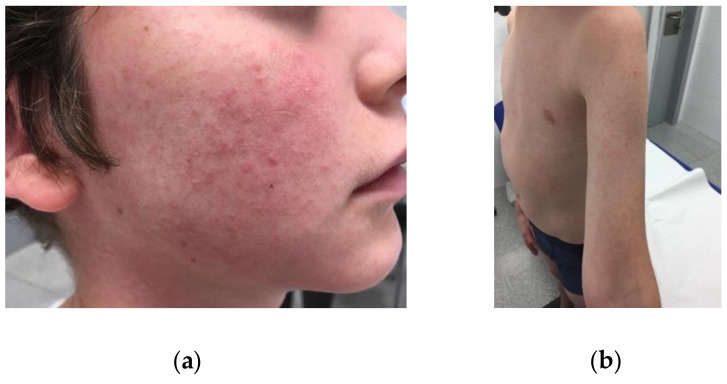
Maculopapular rash on the face (**a**), trunk and arms (**b**) of an 11-year-old boy with vector-borne ZIKV infection.

An 11-year-old boy (case 1) consulted on 9 September 2019 due to a pruritic maculopapular skin rash of one day’s duration (Figure 1). He had traveled with his family through Thailand from 16 August to 6 September 2019, visiting Bangkok, Chiang Mai, and Koh Tao. During the flight back to Barcelona (Catalonia, Spain), he experienced headache, elbow arthralgia, and a low fever that lasted 3 days. Subsequently, a maculopapular rash appeared, and then gradually faded over the next week. To investigate the suspected flavivirus infection, diagnostic tests were performed on 9 September 2019. A real-time reverse transcription–polymerase chain reaction (rRT-PCR) assay for ZIKV (RealStar Zika Virus RT-PCR Kit 1.0; Altona Diagnostics, Germany) yielded positive results in the serum and urine, whereas initial immunoglobulin (Ig) G and IgM tests (anti Zika virus ELISA, IgG and IgM, Euroimmun, Germany) were negative. Serologies and rRT-PCR for dengue virus (DENV) and chikungunya virus (CHKV) were negative. Subsequent serological ZIKV IgG tests showed seroconversion, with negative rRT-PCR results in the serum and urine. Curiously, DENV IgG tested positive in one of the follow-up analyses, but later tests were negative. Hence, the DENV result was considered a false-positive due to the known antibody cross-reactivity when analyzing these viruses (Table 1). None of the other family members travelling at the same time showed signs or symptoms of flavivirus infection, or positive test results.

### 2.2. Family Cluster after Travelling to Thailand

A 33-year-old pregnant woman (case 2) travelled with her 2-year-old daughter (case 3) to Thailand from 17 December 2019 to 14 January 2020. They visited some of the islands of Thailand and the city of Bangkok. The mother reported having been bitten by mosquitoes in a Bangkok hotel on 12 January. Within 2 days, the mother and daughter had both developed a generalized skin rash that lasted 48 h. Neither of them had fever, headache, or conjunctivitis.

After returning from Thailand, the mother sought health care advice on 19 January (4 days after the symptoms onset). Based on the high clinical suspicion, serological and molecular tests for arboviruses in blood (ZIKV, DENV, and CHKV) were performed. ZIKV rRT-PCR and ZIKV IgM tested positive, but CHKV and DENV viruses were negative. Intensive antenatal follow-up was then carried out. Diagnostic testing was done monthly, with successive negative ZIKV rT-PCR results in the urine and serum. ZIKV IgG was first detected 23 days after symptoms onset, and a ZIKV plaque reduction neutralization test (PRNT) was positive. ZIKV IgM remained positive for 2 months (Table 1).

Frequent ultrasound examinations performed during pregnancy showed no fetal abnormalities. At birth, the central nervous system (CNS) ultrasound, automated auditory brainstem response (AABR) tests, and ocular fundus examination in the newborn (case 4) were normal. All ZIKV IgM and rRT-PCR (blood, urine, and saliva) tests were negative in the immediate postnatal period. ZIKV IgG was initially positive, with decreasing titers until seroreversion at 4 months of life. Serologies and rRT-PCR for other flaviviruses were negative (Table 1).

The 2-year-old daughter was seen 17 days after the onset of symptoms. ZIKV IgM, IgG, and PRNT tested positive, but ZIKV rRT-PCR was negative. Serologies and rRT-PCR for CHKV and DENV tested negative (Table 1). She presented no other clinical symptoms apart from a generalized maculopapular rash.

## 3. Discussion

In this report, we describe three cases of vector-borne ZIKV infection in DENV-naïve travelers. Two of them were diagnosed during the viremic period (positive rRT-PCR). We also observed a positive antibody test for DNEV in case 1 at 1 month after the onset of symptoms, suggesting a cross reaction between ZIKV and DENV, as subsequent DENV-IgG tests were negative. In endemic areas for these pathogens, interpretation of serological tests can be highly challenging [1], and PRNT testing can be useful.

The risk assessment for ZIKV infection in pregnant women living in endemic areas depends on the geographic region and the period of study [12,13]. Regarding pregnant travelers, we found no specific risk-related protocols for them when visiting an area where ZIKV is in active circulation. The risk of severe adverse pregnancy outcomes for prenatally exposed babies in these areas is estimated at 5% to 13% [14,15,16,17], with higher rates during the first trimester of pregnancy. However, more information is needed to establish robust recommendations for pregnant women wishing to travel to endemic areas. A recent multicenter cohort study has suggested that the risk of ZIKV infection may be lower in pregnant travelers than in residents, especially when travelling outside epidemic periods, excluding the Caribbean Islands, and staying for less than 2 weeks (RR 0.6, 0.4–0.8) [18].

ZIKV transmission is active in Thailand and other Southeast Asia countries, and the results of several studies indicate that the infection can be transmitted to travelers [8,9,13,19]. The European Centre for Disease Prevention and Control (ECDC) surveillance data of 2018–2019 reported that 50% (13/26) of travel-associated ZIKV infections occurred in people returning from Thailand (10). Therefore, during visits to their clinicians, individuals planning a trip to this country, particularly pregnant women, should be advised about the risk of contracting ZIKV disease. The adult in our series was a dengue-naïve pregnant woman with travel-associated ZIKV infection at the start of the second trimester of pregnancy. On meticulous study, her newborn showed no birth defects potentially related to this condition, although further follow-up is ongoing.

Focusing on the pediatric cases (cases 1 and 3), there are few reports on the imported vector-borne ZIKV infection in dengue-naïve children [5]. A severe disease following the ZIKV infection has rarely been reported in children except for two possible deaths and a small number of Guillain-Barré syndrome and meningoencephalitis cases during the Brazil outbreak in 2015 [11]. In a study conducted in the United States (2015−2016) including 158 confirmed or probable post-travel ZIKV cases in children younger than 18 years, there were no cases of severe disease [20]. These findings corroborate those of previous reports suggesting that the clinical course is typically mild in children [1,5,7,21]. Most pediatric patients only experience a rash [17,20], whereas in adults, complications such as Guillain-Barré syndrome are more frequent. The two cases of vector-borne ZIKV infection in the travelling children reported here were acquired in Thailand. Both patients presented with the maculopapular rash. Neither of them developed any complications or signs of meningitis, encephalitis, or Guillain-Barré syndrome. Nonetheless, we believe that reporting these pediatric ZIKV cases is of value to raise awareness of this disease among pediatricians working in areas where this flavivirus is not endemic.

## 4. Conclusions

Three recent cases of vector-borne ZIKV infection imported from Thailand in a short time period are reported. Fortunately, none of the patients, including a pregnant woman in the second trimester of pregnancy, experienced any adverse outcomes following ZIKV infection. These findings are consistent with other cases of travel-associated ZIKV infection. Clinicians should be aware of the epidemiological risk of acquired vector-borne ZIKV infection in travels to Southeast Asia and adhere to the current WHO recommendations to advise patients of this risk.

## Figures and Tables

**Table 1:** Microbiological Test Results in all Zika Virus (ZIKV) Cases.

**CASE 1**								
***Time since symptoms onset***	**ZIKV**				**DENV**			**CHKV**
	**rRT-PCR Serum** **and Urine**	**IgM**	**IgG**	**PRNT**	**rRT-PCR** **Serum**	**IgM**	**IgG**	**rRT-PCR Serum,** **IgM and IgG**
*4 days*	Positive	Negative	Negative	NT	Negative	Negative	Negative	Negative
*1 month*	Negative	Negative	Positive	NT	Negative	Negative	Positive	Negative
*4 months*	Negative	Negative	Positive	NT	NT	NT	Negative	NT
**CASE 2**								
***Time since symptoms onset***	**ZIKV**					**DENV and CHKV**		
	**rRT-PCR Serum**	**rRT-PCR Urine**	**IgM**	**IgG**	**PRNT**	**rRT-PCR Serum**	**IgM**	**IgG**
*7 days*	Positive	NT	Positive	Negative	NT	Negative	Negative	Negative
*1 month*	Positive	Negative	Positive	Positive	Positive	NT	NT	NT
*2 months*	Negative	NT	Positive	Positive	NT	NT	NT	NT
*3 months*	Negative	Negative	Inconclusive	Positive	NT	NT	NT	NT
*4 months*	Negative	NT	Negative	Positive	NT	NT	NT	NT
*5 months (1 day before labor)*	Negative	NT	Negative	Positive	NT	NT	NT	NT
**CASE 3**								
***Time since symptoms onset***	**ZIKV**					**DENV and CHKV**		
	**rRT-PCR Serum**	**rRT-PCR Urine**	**IgM**	**IgG**	**PRNT**	**rRT-PCR Serum**	**IgM**	**IgG**
*17 days*	Negative	Negative	Positive	Positive	Positive	Negative	Negative	Negative
**CASE 4**								
***Age***	**ZIKV**					**DENV and CHKV**		
	**rRT-PCR Serum**	**rRT-PCR Urine**	**IgM**	**IgG**	**PRNT**	**rRT-PCR Serum**	**IgM**	**IgG**
*1 day*	Negative	Negative	Negative	Positive	NT	NT	NT	NT
*1 month*	Negative	NT	Negative	Positive	NT	Negative	Negative	Negative
*4 months*	Negative	NT	Negative	Negative	NT	Negative	Negative	Negative

ZIKV: Zika virus; DENV: Dengue virus; CHKV: Chikungnya virus; rRT-PCR: Real-time reverse transcription–polymerase chain reaction; PRNT: Plaque reduction neutralization test; NT: Not tested.

## Data Availability

No new data were created or analyzed in this study. Data sharing is not applicable to this article.
